# Supplementary material for: Benefits and harms of copyright restrictions and conditions on burnout and other psychometric assessment scales
Source: PLoS One. 2026 May 21;21(5):e0350023. doi: 10.1371/journal.pone.0350023 (PMC13193556; doi:10.1371/journal.pone.0350023)
Supplement: S1 Table — (DOCX) [file pone.0350023.s001.docx]

**Supporting information for: Benefits and harms of copyright restrictions and conditions on burnout and other psychometric assessment scales**

| **Table. Search terms used for MEDLINE for each scale.** | | | |
| --- | --- | --- | --- |
| Scale | Original citation (PMID) | Search terms for numerator:  Scale name* | Search terms for denominator: setting of initial use of the scale† |
| PHQ-9 | 10568646 | ‘PHQ-9'[TIAB] | ‘Depressive disorder'[MeSH] |
| SLUMS | 17068312 | ‘Saint Louis University Mental Status'[TIAB] | Dementia[TIAB] |
| MBI | NA | ‘Maslach Burnout'[TIAB] | Burnout[TIAB] |
| MMSE | 1202204 | ‘Mini-mental status'[TIAB] | Dementia[TIAB] |
| APGAR | 13083014 | APGAR[TIAB] | Newborn*[ALL] |
| CAGE | 4416585 | CAGE Question*[TIAB] | ‘Alcohol Problem’[MeSH] |
| **Notes**:  * TIAB. Title or abstract.  † Search terms for the setting were chosen for the initial setting for which the scale was developed. This allowed the identification of possible new settings where the survey or scale was being applied. | | | |

[rbadgett@kumc.edu](mailto:rbadgett@kumc.edu)

<https://ebmgt.github.io/copyright_case_studies>

2026-04-10
