# Supplementary material for: Benefits and harms of copyright restrictions and conditions on burnout and other psychometric assessment scales
Source: PLoS One. 2026 May 21;21(5):e0350023. doi: 10.1371/journal.pone.0350023 (PMC13193556; doi:10.1371/journal.pone.0350023)
Supplement: S1 Figure — (DOCX) [file pone.0350023.s002.docx]

**Supporting information for: Benefits and harms of copyright restrictions and conditions on burnout and other psychometric assessment scales**

**Figure.** Example using the Maslach Burnout Inventory to show the calculation of the citation ratio and its resulting plot.

**
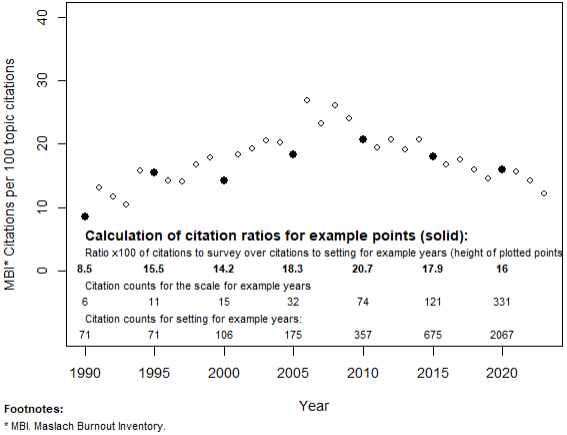
**

[rbadgett@kumc.edu](mailto:rbadgett@kumc.edu)

<https://ebmgt.github.io/copyright_case_studies>

2025-12-07
