# Supplementary material for: Benefits and harms of copyright restrictions and conditions on burnout and other psychometric assessment scales
Source: PLoS One. 2026 May 21;21(5):e0350023. doi: 10.1371/journal.pone.0350023 (PMC13193556; doi:10.1371/journal.pone.0350023)
Supplement: S2 Figure — (DOCX) [file pone.0350023.s003.docx]

**Supporting information for: Benefits and harms of copyright restrictions and conditions on burnout and other psychometric assessment scales**

**Figure.** Example using the Maslach Burnout Inventory to show the selection process to identify the optimal number of knots (deflections) in the regression plots. The shaded row indicates the optimal number of knots.

| Plot thumbnail | Knots (interior) | R^2^ | P-value compared to one fewer knot |
| --- | --- | --- | --- |
| 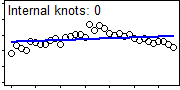 | 0  (linear regression) | 0.038 | 0.139 |
| 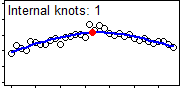 | 1 | 0.758 | 0.000 |
| 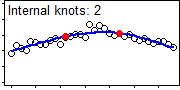 | 2 | 0.749 | NA |
| 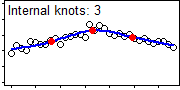 | 3 | 0.793 | 0.011 |
| 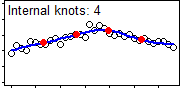 | 4 | 0.784 | NA |
| 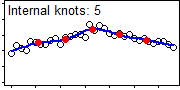 | 5 | 0.817 | 0.021 |
| The remaining plots insignificantly add fit and are not shown. | 6 through 9 | < 0.805 | NA |
| * NA. There was insufficient difference between the models to calculate a P value. | | | |
