# Supplementary material for: Benefits and harms of copyright restrictions and conditions on burnout and other psychometric assessment scales
Source: PLoS One. 2026 May 21;21(5):e0350023. doi: 10.1371/journal.pone.0350023 (PMC13193556; doi:10.1371/journal.pone.0350023)
Supplement: S3 Figure — (DOCX) [file pone.0350023.s004.docx]

**Supporting information for: Benefits and harms of copyright restrictions and conditions on burnout and other psychometric assessment scales**

**Figure.** Two surveys/scales with permissive copyrights and long-term follow-up possible.

| **Panel A.** The APGAR score.  **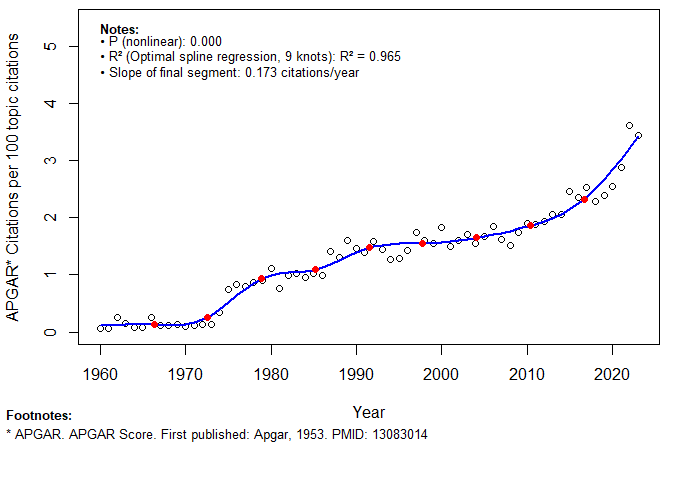** |
| --- |
| **Panel B.** The CAGE Questionnaire.  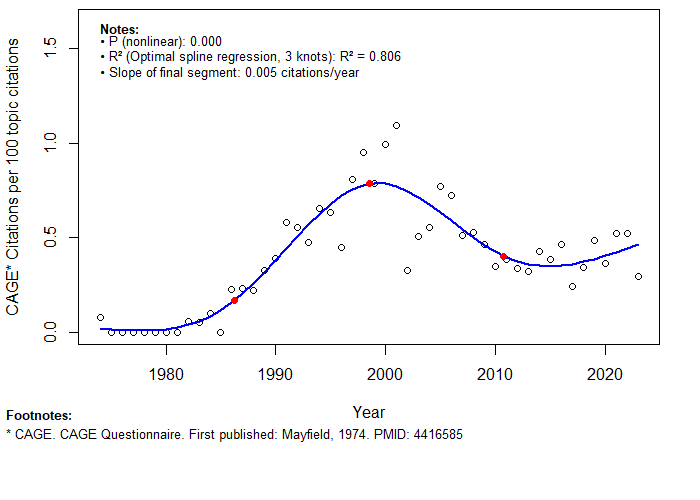 |
