# Supplementary material for: Benefits and harms of copyright restrictions and conditions on burnout and other psychometric assessment scales
Source: PLoS One. 2026 May 21;21(5):e0350023. doi: 10.1371/journal.pone.0350023 (PMC13193556; doi:10.1371/journal.pone.0350023)
Supplement: S1 Text — (DOCX) [file pone.0350023.s005.docx]

**Supporting information for: Benefits and harms of copyright restrictions and conditions on burnout and other psychometric assessment scales**

**Publishing a survey with a blended license at the Open Science Framework**

The Creative Commons (CC) gives two resources for learning about licensing options. The first is a list of options with short descriptions and the second is an interactive dialogue to help choose an option.

- <https://creativecommons.org/cclicenses/>
- <https://creativecommons.org/choose>

The Open Science Framework provides resources to learn about hosting projects or preprints on their website

- <https://help.osf.io/>

[rbadgett@kumc.edu](mailto:rbadgett@kumc.edu)

<https://ebmgt.github.io/copyright_case_studies>

2026-04-11
